# Roles of the *spiA* gene from *Salmonella enteritidis* in biofilm formation and virulence

**DOI:** 10.1099/mic.0.046185-0

**Published:** 2011-06

**Authors:** Hongyan Dong, Daxin Peng, Xinan Jiao, Xiaorong Zhang, Shizhong Geng, Xiufan Liu

**Affiliations:** 1 College of Veterinary Medicine, Ministry of Education Key Lab for Avian Preventive Medicine, Yangzhou University, Yangzhou, Jiangsu 225009, PR China; 2 Jiangsu Key Laboratory of Zoonosis, Yangzhou University, Yangzhou, Jiangsu 225009, PR China

## Abstract

*Salmonella enteritidis* has emerged as one of the most important food-borne pathogens for humans, and the formation of biofilms by this species may improve its resistance to disadvantageous conditions. The *spiA* gene of *Salmonella typhimurium* is essential for its virulence in host cells. However, the roles of the *spiA* gene in biofilm formation and virulence of *S. enteritidis* remain unclear. In this study we constructed a *spiA* gene mutant with a suicide plasmid. Phenotypic and biological analysis revealed that the mutant was similar to the wild-type strain in growth rate, morphology, and adherence to and invasion of epithelial cells. However, the mutant showed reduced biofilm formation in a quantitative microtitre assay and by scanning electron microscopy, and significantly decreased curli production and intracellular proliferation of macrophages during the biofilm phase. In addition, the *spiA* mutant was attenuated in a mouse model in both the exponential growth and biofilm phases. These data indicate that the *spiA* gene is involved in both biofilm formation and virulence of *S. enteritidis*.

## Introduction

*Salmonella enterica* serovar Enteritidis (*Salmonella enteritidis*) has emerged as one of the most important food-borne pathogens for humans; it is mainly associated with consumption of contaminated poultry meat and eggs (Patrick *et al.*, 2004). A number of studies have demonstrated that *Salmonella* bacteria are capable of forming biofilms on a wide variety of contact surfaces (Korber *et al.*, 1997; Römling *et al.*, 2003), and the formation of biofilms may improve the ability of these organisms to resist stresses such as desiccation, extreme temperatures, antibiotics and antiseptics (Marin *et al.*, 2009; Scher *et al.*, 2005). Biofilm formation allows *Salmonella* to survive long term in the poultry farm environment and to contaminate poultry meat and eggs, which remain the leading vehicles of food-borne salmonellosis outbreaks (Joseph *et al.*, 2001).

In recent years, many groups have dedicated great effort to identifying the factors involved in biofilm development. Curli and cellulose are the major components of biofilm formation in *Salmonella* (Römling *et al.*, 1998b; White *et al.*, 2003; Zogaj *et al.*, 2001); capsular polysaccharide (de Rezende *et al.*, 2005), other polysaccharides such as LPS (Anriany *et al.*, 2006) and a large secreted protein, BapA, also contribute to biofilm formation (Latasa *et al.*, 2005). Several regulatory genes are involved in biofilm formation. For example, the *csgD* gene encodes a transcriptional regulator of the LuxR superfamily and has been shown to critically control expression of curli and cellulose (Gerstel & Römling, 2003), which are positively regulated by the *ompR* and *rpoS* genes (Römling *et al.*, 1998a). Hamilton *et al.* (2009) determined the transcriptomic and proteomic profiles of *Salmonella typhimurium* biofilms and found that a functional SPI2 secretion system regulator (*ssrA*), an STM0341 gene of unknown function, and a *trpE* gene that is involved in tryptophan biosynthesis and transport were needed for biofilm growth. Recently, we identified several genes associated with biofilm formation by transposon mutagenesis in *Salmonella* (Dong *et al.*, 2008). However, the detailed roles of some of the genes identified to be involved in biofilm formation remain unclear. In this study, the roles of the *spiA* gene in biofilm formation and virulence were investigated.

## Methods

### 

#### Strains, plasmids and growth conditions.

The bacterial strains and plasmids used in this study are listed in Table 1. Bacteria were grown in rich liquid or solid (15 g agar l^−1^) Luria broth (LB) medium with antibiotic supplementation as needed, such as ampicillin (Amp, 100 µg ml^−1^), kanamycin (Km, 25 µg ml^−1^) and streptomycin (Sm, 100 µg ml^−1^). Nutrient agar (NA, solid LB medium without NaCl) and nutrient broth (NB, liquid LB medium without NaCl) with 10 % sucrose were used during the allelic-exchange experiments. Growth rates of the wild-type strain and the mutant were measured as follows. An overnight culture was inoculated in 10 ml LB medium (adjusted to OD_600_ 0.1) and shaken at 200 r.p.m. at 37 °C. The bacterial cultures were monitored spectrophotometrically at 600 nm every hour for 7 h. For preparation of bacteria in the exponential phase of growth, the bacteria were streaked on LB agar, incubated at 37 °C for 4–5 h, washed twice with PBS and suspended in PBS. For preparation of bacteria in biofilm growth, overnight broth cultures were diluted 1 : 10 in cell culture flasks and incubated at 28 °C for 48 h. The biofilm cells that formed on flasks were dislodged by vigorous vortexing with glass beads in PBS and then resuspended in PBS.

**Table 1.** Summary of the strains, plasmids and primers used

Strain, plasmid or primer	Description/purpose	Reference or source
***E. coli* strains**		
C50041	Wild-type strain	Qiuchun *et al.* (2009)
χ7213	For cloning pGMB151	Kang *et al.* (2002)
S17-λ*pir* (Sm^R^)	For cloning pGMB151	Huang *et al.* (2004)
Δ*spiA*	*spiA* mutant	This study
Δ*spiA*R	Complementation of Δ*spiA*	This study
**Plasmids**		
pUC18(Amp^r^)	Plasmid for cloning	Promega
pMD18-T	Plasmid for cloning	Takara
pGMB151(Amp^r^ Sm^r^)	Suicide plasmid	Huang *et al.* (2004)
pBluescript II SK(−)	Plasmid for cloning	Fermentas
pUC4K	Plasmid for kanamycin-resistance gene	Amersham
pGEX-6P-1	Plasmid for *spiA* gene restoration and *csgA* gene expression	Amersham
**Primers**		
12	5′-CTCGGATCCCACTATGTACTTGAGTCGTATCATTGCG-3′ (*spiA* sense, *Bam*HI site underlined)	This study
13	5′-CTCGTCGACCCCTTTATGCCAGACAAATGCCA-3′ (*spiA* antisense, *Sal*I site underlined)	This study
14	5′-CTCGAATTCGCGTAAGTAATGAATGAACCGTCC-3′ [pBluescript II SK(−)+*spiA*1 sense, *Eco*RI site underlined]	This study
15	5′-CTCGAATTCCGGGTTCTTTGTTGCGCGTT-3′ [pBluescript II SK(−)+*spiA*2 antisense, *Eco*RI site underlined]	This study
16	5′-CAGTGACAGGTTACCTTCATTCAGC-3′ (*spiA* sense, *spiA* design primer)	This study
17	5′-CTCGGATCCCCCTTTATGCCAGACAAATGCCA-3′ (*spiA* antisense, *Bam*HI site underlined)	This study
18	5′-GAATTCTCAAAGCCACGTTGTGTCTCAAA-3′ (kanamycin sense, *Eco*RI site underlined)	This study
19	5′-GAATTCCGCTGAGGTCTGCCTCGT-3′ (kanamycin antisense, *Eco*RI site underlined)	This study
20	5′-TGCAAGTTAAAGCCAGGTG-3′ (*spiC* sense)	This study
21	5′-CCGAAGGTAATAGCCGATCC-3′ (*spiC* antisense)	This study
22	5′-GAGGTATCGCTGATTACCGTTG-3′ (*spiA* sense)	This study
23	5′-CGTGAGTGTATTGCGTAGGATG-3′ (*spiA* antisense)	This study
24	5′-CACTGGCGCAGGCAGATAC-3′ (*ssaD* sense)	This study
25	5′-GATCAGCAATGGCGTAAGGTTC-3′ (*ssaD* antisense)	This study
26	5′-CTCGGATCCATGGTAGTAAATAAACGTTTAATC-3′ (*spiA* complementation sense, *Bam*HI site underlined)	This study
27	5′-CTCCTCGAGTTAACCATGAGATATGCCATTATTTAC-3′ (*spiA* complementation antisense, *Xho*I site underlined)	This study

#### Construction of the suicide plasmid, pGMB151-*spiA*-Kan.

The *spiA* gene was amplified from the chromosomal DNA from *S. enteritidis* strain C50041 by using primers 12/13 (see Table 1 for sequences of all primers used). The PCR product was purified and cloned into a pSK(−) vector to obtain pSK-*spiA*. The SK-*spiA* fragment was amplified from pSK-*spiA* by using reverse PCR with primers 14/15. The fragment was then ligated to a kanamycin-resistance cassette (Kan) amplified from pUC4K with primers 18/19 to produce pSK-*spiA*-Kan, which resulted in insertion of the kanamycin gene into the *spiA* gene with a 767 bp deletion. The PCR product of *spiA*-Kan, amplified from pSK-*spiA*-Kan with primers 12 /17, was digested with *Bam*HI and subsequently inserted into the *Bam*HI site of the suicide plasmid, pGMB151, to form pGMB151-*spiA*-Kan. After verification by sequence analysis, the resulting plasmid was transferred to *Escherichia coli* S17-λpir (Sm^r^) and further transferred to *E. coli *χ7213 as donor strain *E. coli* χ7213(pGMB151-*spiA*-Kan).

#### Construction of a *spiA* mutant by allelic exchange and complementation of *spiA*.

The donor strain, *E. coli *χ7213(pGMB151-*spiA*-Kan), and the recipient strain, C50041, were grown in LB containing 1 % diaminopimelic acid with shaking at 37 °C to an OD_600_ of 0.6–0.7. The donor strain and the heat-treated recipient strain were mixed in 10 mM MgSO_4_, the mixture was immobilized on a 0.45 µm membrane filter and the filter was incubated on LB agar at 30 °C for 18 h. The transconjugants were spread onto LB agar containing Amp, Sm and Km. Colonies were then streaked onto NA with Amp, Sm, Km and sucrose to select bacteria that were sensitive to sucrose (Geng *et al.*, 2009). A single bacterial colony that was sensitive to sucrose was subcultured 8–10 times on NB containing 10 mM MgSO_4_, 10 % sucrose and Km. The Km^r^ mutants without Amp^r^ and Sm^r^ were screened on LB plates containing Km. Finally, the *spiA* mutant was confirmed by primers 13/16 and named Δ*spiA.* For gene complementation, *spiA* was amplified from the C50041 strain with primers 26/27. The PCR product was cloned into pGEX-6P-1, and the ligation mixture was transformed into *E. coli* BL21. Plasmid p6P-*spiA* was isolated from the Amp^r^ transformants, and the presence of an insert was verified by restriction analysis and sequence analysis. Plasmid p6P-*spiA* was transformed into Δ*spiA* to create a *spiA*-complemented strain, Δ*spiA*R.

#### RT-PCR.

Total RNA was isolated from exponential phase bacteria of strains C50041 and Δ*spiA* by the hot phenol method. The RNA was treated with DNase I (Takara) and used as a template for reverse transcription (RT) with random primers. The primer sets for cDNA amplification of target genes *spiC*, *spiA* and *ssaD* are given in Table 1. The PCR products were resolved on 1 % agarose gels and visualized by ethidium bromide staining.

#### SDS-PAGE.

A single colony each of Δ*spiA* and C50041 was inoculated into 10 ml LB and shaken at 37 °C for 14 h. The supernatant was collected by centrifugation at 12 000 r.p.m. for 1 min and put on ice for approximately 30 min after the addition of 1 ml trichloroacetic acid. The proteins were harvested by centrifugation at 12 000 r.p.m. for 10 min, washed twice with 5 ml acetone, dried at 4 °C, dissolved in 50 µl PBS and examined by SDS-PAGE on a 10 % separating gel with 40 µg protein.

#### Biofilm formation.

A quantitative microtitre assay was performed as described by Pratt & Kolter (1998). The morphology of the biofilm formed was also determined by scanning electron microscopy as described by Anriany *et al.* (2001), with minor modifications. Strains Δ*spiA* and C50041 were inoculated in 3 ml LB in a six-well cell-culture plate containing polystyrene coverslips (0.5 cm×0.5 cm) and incubated at 28 °C for 48 h without shaking. The slips with bacterial cells were removed, fixed in 3 % glutaraldehyde-PBS (pH 7.0) for 2 h, gently washed three times with PBS (pH 7.0), and dehydrated through graded ethanol/water mixtures with 30 % (15 min), 50 % (15 min), 70 % (15 min), 80 % (15 min), 90 % (15 min) and 100 % (15 min×3) ethanol. Dried chips were then immersed in ethanol/isoamyl acetate (1 : 1 and 2 : 1) for 30 min and then transferred to isoamyl acetate for 30 min. They were dried by critical-point drying, coated with gold–palladium alloy and observed with a scanning electron microscope (S4800; Hitachi).

#### Determination of curli and cellulose.

To determine whether Δ*spiA* produced curli and/or cellulose, 10 µl samples of Δ*spiA* and the wild-type strain, C50041, cultured overnight, were dropped on LB agar lacking NaCl, supplemented with Congo red (40 µg ml^−1^; Sigma) and Coomassie brillliant blue G (20 µg ml^−1^; Sigma), and incubated at 28 °C for 48 h. The morphology was then examined. In a separate experiment, production of cellulose by Δ*spiA* and C50041 was determined by inoculating 10 µl samples on NA supplemented with 200 mg Calcofluor ml^−1^ (fluorescent brightener 28; Sigma) at 28 °C for 48 h, and comparing the fluorescence of Δ*spiA* and C50041 under UV light (366 nm). Production of curli protein was determined as described by Anriany *et al.* (2006). One millilitre of suspension with Δ*spiA*, C50041 and Δ*spiA*R in the biofilm phase (OD_600_ 3.0) was centrifuged, and the cell pellets were resuspended in 100 µl SDS sample buffer [62.5 mM Tris/HCl (pH 6.8), 10 % glycerol, 2 % SDS] and boiled for 10 min. The cell lysate was centrifuged, and the pellet was washed twice with sterile water, dissolved in 100 µl 97 % formic acid (Sigma), frozen at −70 °C and lyophilized. The samples were resuspended in 100 µl SDS sample buffer, sonicated for 5 s and examined by SDS-PAGE. Separated proteins were transferred onto a nitrocellulose membrane. Western blotting was performed using mouse antisera specific against the curli major subunit, CsgA, which was prepared in mice immunized with an expression product of pGEX-6P-1-*csgA.*

#### Adherence and invasion assay.

The A549 human epithelial cell line was cultured in RPMI 1640 with 10 % fetal calf serum at 37 °C in 5 % CO_2_ (Monack *et al.*, 1996). Briefly, a 1 ml portion with 1×10^5^ cells was seeded into each well of 24-well plates and incubated for 16 h. Cell cultures of strains Δ*spiA* and C50041 in the exponential and biofilm phase of growth were suspended in PBS and adjusted to OD_600_ 0.4. The mean concentrations of strains C50041 and Δ*spiA* in exponential phase growth were 3.6×10^8^ and 3.8×10^8^ c.f.u. ml^−1^, respectively, while the concentrations in the biofilm phase were approximately 4.5×10^8^ and 4.8×10^8^ c.f.u. ml^−1^, respectively, according to viable bacteria counting. Suspensions with an m.o.i. of 100 were inoculated in duplicate in a 24-well plate. This plate was centrifuged for 5 min at 100 ***g*** and then incubated at 37 °C for 2 h, the wells were rinsed three times with PBS, and the cells were released from the plate by adding 0.05 % trypsin. For the invasion assay, the cells were further cultured with 100 µg gentamicin ml^−1^ for 1 h and lysed with 1 % Triton X-100. Both cell suspensions were serially diluted in PBS and spread onto LB plates to determine the number of viable bacteria. Adherence and invasion were expressed as the percentage of bacteria that were attached and that invaded the cells relative to the original number of bacteria added to the well. The experiments were repeated in three independent assays.

#### Intracellular growth rate assay.

For this assay, a mouse macrophage cell line, RAW264.7, was used and the method was the same as that used for the adherence assay. Cell cultures of strains Δ*spiA* and C50041 in the exponential and biofilm phases were diluted in PBS to allow for infection at an m.o.i. of 10. Infection cultures were incubated for 2 h, rinsed three times in PBS and incubated with 100 µg gentamicin ml^−1^ for 1 h. The intracellular bacteria were counted as above. For determination of intracellular bacterial proliferation, cells were incubated for an additional 20 h with 10 µg gentamicin ml^−1^. The experiments were repeated in three independent assays.

#### Determination of LD_50_ in mice.

BALB/c mice (6 weeks of age) were obtained from the experimental animal centre of Yangzhou University. The mice were housed in an animal facility under a standard animal study protocol. Four separate groups of mice (each containing 30 mice) were infected with Δ*spiA* and C50041 in the exponential and biofilm phases. Mice in each group were further subdivided into five subgroups, each containing six mice. Based on the results of a pilot study, each mouse in the Δ*spiA* groups was injected intraperitoneally with 0.2 ml of 2×10^8^, 2×10^7^, 2×10^6^, 2×10^5^ and 2×10^4^ c.f.u., respectively, and each mouse in the C50041 groups was injected intraperitoneally with 0.2 ml of 2×10^4^, 2×10^3^, 2×10^2^, 2×10^1^ and 2×10^0^ c.f.u., respectively. Deaths were recorded up to day 14, and the LD_50_ of each strain was calculated by the method of Reed & Muench (1938).

#### Distribution of Δ*spiA* and C50041 in mice.

Strains Δ*spiA* and C50041 in the exponential and biofilm phases were suspended in PBS and adjusted to 10^7^ c.f.u. ml^−1^. Six-week-old BALB/c mice were divided into four groups (five per group) and each mouse was administered intraperitoneally with a 0.2 ml bacterial suspension. The numbers of bacteria present in the blood, liver, spleen and lungs of the mice were measured 6 and 48 h post-challenge.

#### Statistical analysis.

The numbers of viable bacteria (c.f.u.) in mice were expressed as the geometric mean±sd. Significance was analysed by using a two-tailed independent Student’s *t*-test. The adherence, invasion and intracellular growth percentages were analysed by chi-squared tests.

## Results

### Construction and identification of the Δ*spiA* mutant

The *spiA* gene of strain C50041 was amplified with primers 12/13 and cloned into pMD18-T. Nucleotide sequence analysis showed that this DNA fragment contained a single open reading frame of 1494 bp. A Δ*spiA* mutant was constructed by allelic exchange with a substitution of a kanamycin-resistance cassette and a 767 bp deletion within the *spiA* coding region. A 1.93 kb fragment amplified with primers 16/13 identified the Δ*spiA* mutation including the kanamycin-resistance cassette. Sequence analysis of the PCR product confirmed that the kanamycin-resistance cassette had been inserted into *spiA* of C50041 chromosomal DNA at the predicted position.

To determine whether the insertion had a polar effect on the downstream gene, total RNA isolated from both Δ*spiA* and C50041 was subjected to RT-PCR analysis using primer sets for *spiC* (primers 20/21), *spiA* (primers 22/23), and *ssaD* (primers 24/25). Compared with the wild-type strain, C50041, insertion of the kanamycin resistance cassette in Δ*spiA* disrupted only the transcription of the *spiA* gene and had no effect on transcription of the upstream *spiC* gene or the downstream *ssaD* gene (data not shown).

### Growth rate and protein pattern of Δ*spiA*

**Fig. 1.**
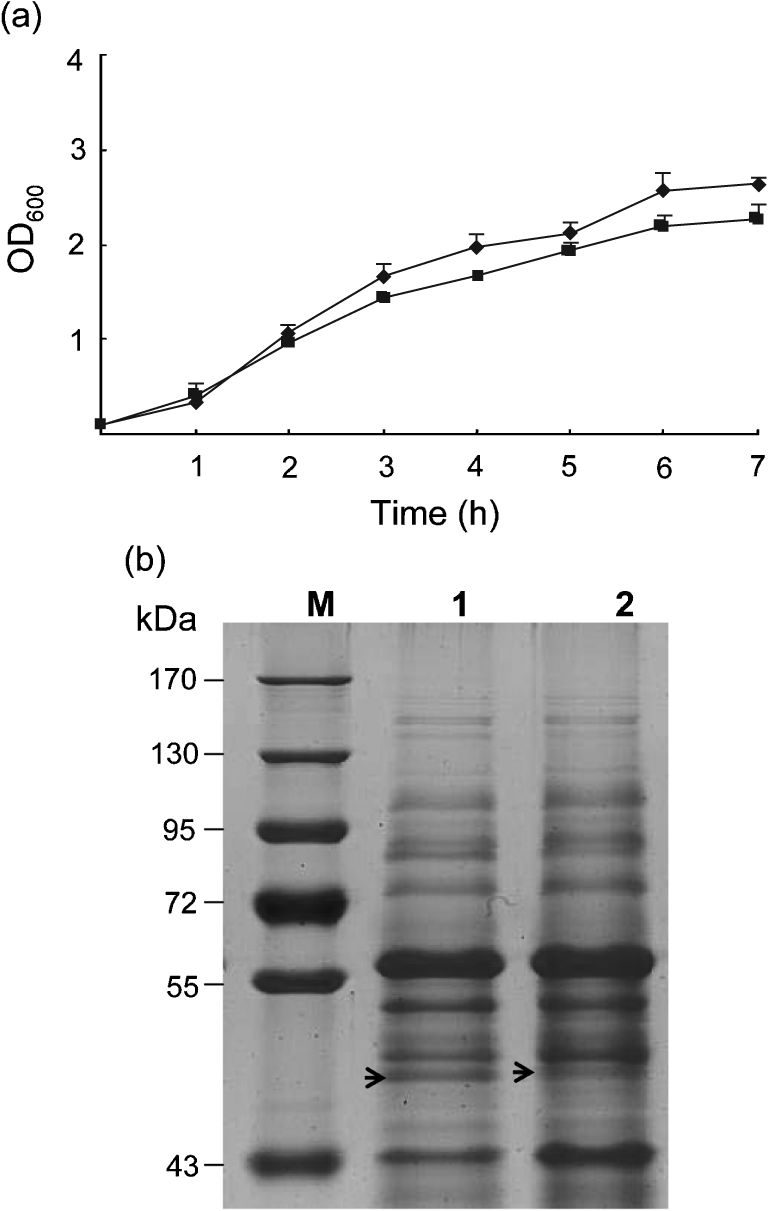
Growth curves and protein patterns of the wild-type strain, C50041, and the *spiA* mutant, Δ*spiA*. (a) Cultures of C50041 (⧫) and Δ*spiA* (▪) were monitored spectrophotometrically at 600 nm hourly for 7 h. (b) Proteins (40 µg samples) prepared from the supernatants of C50041 (lane 1) and Δ*spiA* (lane 2) cultures were subjected to SDS-PAGE. Prestained protein markers are indicated on the left (lane M).

When grown in LB, the growth rate of Δ*spiA* was similar to that of C50041 in the exponential phase of growth (*P*>0.05; Fig. 1a). Comparative analysis of the protein profile indicated that a 45 kDa protein appeared in the supernatant of C50041, while a 47 kDa protein appeared in that of Δ*spiA* (Fig. 1b). Mass spectrometry analysis (Fitgene, China) showed that both proteins included flagellin peptide residues, which is a similar protein pattern to that described by Ochman *et al.* (1996).

### Biofilm formation by Δ*spiA*

**Fig. 2.**
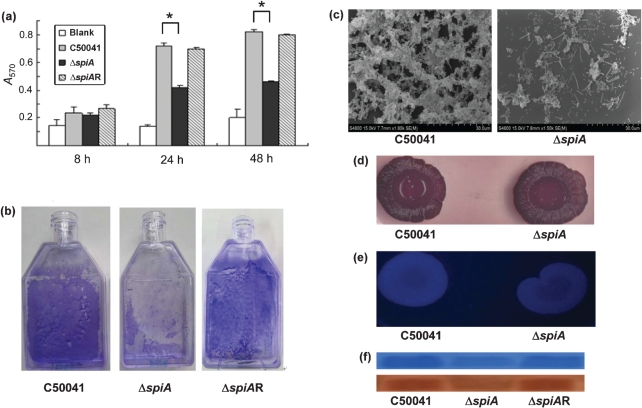
Determination of biofilm formation and its components in the wild-type strain (C50041), the *spiA* mutant (Δ*spiA*) and the *spiA*-complemented strain (Δ*spiA*R). (a) *A*_570_ values of crystal-violet-stained C50041, Δ*spiA* and Δ*spiA*R biofilms grown in 96-well plates. (b) Overnight broth cultures were diluted 1 : 10 in cell-culture flasks. After incubation at 28 °C for 48 h, the broth was removed and the biofilm cells were stained with crystal violet. (c) Scanning electron micrographs of cells of the wild-type strain C50041 and Δ*spiA* grown on polystyrene coverslips at 28 °C for 48 h. (d, e) Morphotypes of C50041 and Δ*spiA* grown on plates of LB agar lacking NaCl and supplemented with Congo red and Coomassie brilliant blue G (d) or with Calcofluor (e). (f) Biofilm cells of strains C50041, Δ*spiA* and Δ*spiA*R with the same optical densities (OD_600_ 3.0) were collected and treated. Curli proteins were examined by SDS-PAGE with 15 % separating gel (top) and confirmed by Western blotting (bottom).

To investigate the effect of the loss of a functional *spiA* gene on biofilm formation, Δ*spiA* was tested for biofilm-associated biological activity. In a quantitative microtitre assay, the *A*OD value of Δ*spiA* was significantly lower than that of the wild-type strain, C50041, at 24 and 48 h post-inoculation (Fig. 2a). When cultured in a flask, both Δ*spiA* and C50041 formed biofilms on the flask wall, but the biofilm of Δ*spiA* was less adherent than that of C50041 (Fig. 2b). In contrast, a *spiA* complementation strain, Δ*spiA*R, regained the same biofilm characteristics as the wild-type strain (Fig. 2a, b). Scanning electron microscopy of biofilm formation by the strains showed that Δ*spiA* produced a loose and thin structure with fewer bacteria, while C50041 produced a very tenacious and thick structure with extracellular substances and fibrils that seemed to bind the cells together (Fig. 2c).

Because biofilms of *Salmonella* are mainly composed of cellulose and curli (Römling *et al.*, 1998b; White *et al.*, 2003; Zogaj *et al.*, 2001), Δ*spiA* was tested on LB agar lacking NaCl and supplemented with Congo red and Coomassie brilliant blue. Strain Δ*spiA* formed red, dry and rough colonies, similar to those of the wild-type strain (Fig. 2d). The presence of cellulose was further tested with Calcofluor. The fluorescence of Δ*spiA* was similar in brightness to that of C50041 (Fig. 2e), indicating that Δ*spiA* still produced cellulose. Comparative analysis of curli proteins by SDS-PAGE showed that both strains produced a 15.3 kDa band (the major subunit of curli protein; Fig. 2f). However, the intensity of the band produced by Δ*spiA* was between 47.0 and 60.5 % of that produced by C50041, according to the Quantity One software (Bio-Rad), indicating that Δ*spiA* produced fewer curli. SDS-PAGE and Western blotting confirmed that the Δ*spiA*R regained a similar level of expression of the curli protein as the wild-type strain.

### Biological activity of Δ*spiA*

To test the adherence and invasion of Δ*spiA* of A549 human epithelial cells, bacteria in both the exponential and the biofilm phases of growth were used (Table 2). The percentages of adherence and invasion of A549 cells by Δ*spiA* in the biofilm phase were higher than those in the exponential growth phase. Lack of expression of the *spiA* gene caused no reduction in adherence to and invasion of A549 cell lines compared with those of the wild-type strain.

**Table 2.** Adherence to and invasion of A549 cells by strains C50041 and Δ*spiA* Values are mean±sd.

Strain	Growth phase	Percentage adherence (no. adhered/no. inoculated)	Percentage invasion (no. invaded/no. inoculated)
C50041	Exponential	0.40±0.20	0.40±0.01
Δ*spiA*		0.59±0.09	0.63±0.13
C50041	Biofilm	25.7±2.2	3.5±0.01
Δ*spiA*		25.5±4.2	3.7±0.50

To test the proliferation capacity of Δ*spiA* in mouse macrophage-like RAW264.7 cells, both bacteria in the exponential growth and biofilm phases were used (Table 3). For bacteria in the exponential growth phase, the invasion and proliferation ratios of the two strains were similar at 3 and 23 h post-infection. However, for bacteria in the biofilm phase, the proliferation ratio of Δ*spiA* increased 24.7-fold from 3 to 23 h post-infection, which was significantly lower than that of the wild-type strain (44.29-fold; *P*<0.05). When comparing the two strains in different conditions, strains in the biofilm phase were more invasive than those in the exponential growth phase.

**Table 3.** Intramacrophage survival properties of strains C50041 and Δ*spiA* Values are mean±sd.

Strain	Growth phase	Percentage invasion (3 h)	Percentage proliferation (23 h)	Proliferation ratio (23 h/3 h)
C50041	Exponential	1.43±0.25	35±7.1	24.5±0.71
Δ*spiA*		1.15±0.21	34±4.9	29.23±1.09
C50041	Biofilm	29±3.5	1273±3.22	44.29±5.8*
Δ*spiA*		46±6	1140±3.39	24.7±3.98

* The proliferation rate of strain C50041 was significantly greater than that of Δ*spiA* in the biofilm phase (*P*<0.05).

**Fig. 3.**
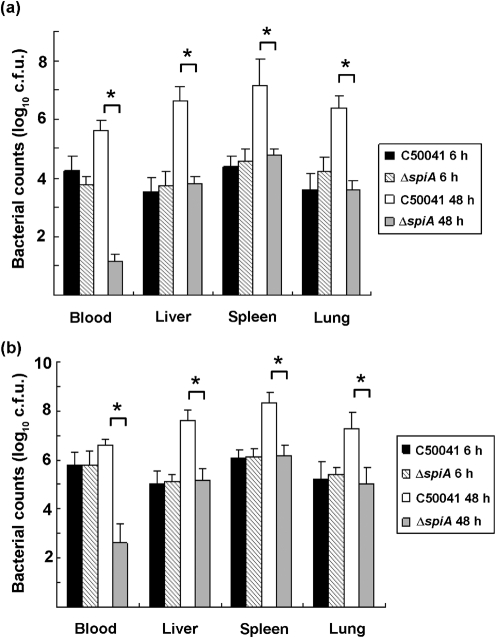
Distribution of bacteria in BALB/c mice challenged with exponential-phase cells (a) or biofilm-phase cells (b) of the wild-type strain C50041 and the *spiA* mutant Δ*spiA*. Six-week-old BALB/c mice were intraperitoneally injected with 0.2 ml of bacterial suspension. The numbers of bacteria present in the blood, liver, spleen and lungs of the mice were measured 6 and 48 h post-challenge. Significant differences in the bacterial counts of organs were determined by Student’s *t*-test. *, *P*<0.05.

To investigate the effect on the virulence of Δ*spiA*, mice were injected intraperitoneally with Δ*spiA* and C50041, and LD_50_ values were calculated according to the method of Reed & Muench (1938). LD_50_ values of C50041 in the biofilm and exponential growth phases were 10^−0.03^ and 10^0.13^, respectively, and those of Δ*spiA* were 10^7.47^ and 10^7.13^, respectively. To further investigate the distribution of bacteria *in vivo*, BALB/c mice were injected intraperitoneally with 2×10^6^ c.f.u. and then killed at 6 and 48 h post-infection. At 6 h post-challenge with bacteria in the exponential growth phase, the numbers of Δ*spiA* present in the blood, liver, spleen and lungs were similar to those of C50041. At 48 h post-challenge, the numbers of Δ*spiA* in blood, liver, spleen and lungs were significantly lower than those of the wild-type strain, C50041 (Fig. 3a). The distribution pattern in the organs of mice challenged with bacteria in the biofilm phase was similar to that with bacteria in the exponential growth phase. However, bacterial counts in organs challenged with bacteria in the biofilm phase were significantly higher than with bacteria in the exponential growth phase, except for the counts in blood at 6 h post-challenge (Fig. 3b). In addition, the clearance ratio in blood with Δ*spiA* in the exponential growth phase decreased 412-fold from 6 to 48 h post-infection. By contrast, the clearance ratio in blood with Δ*spiA* in the biofilm phase decreased 1548-fold from 6 to 48 h post-infection, a 3.75-fold greater clearance rate.

## Discussion

The *spiA* gene within *S. typhimurium*, which encodes an outer-membrane component of the SPI-2 type III secretion system, is essential for virulence in host cells (Ochman *et al.*, 1996). It has also been demonstrated by transposon mutagenesis that the *spiA* gene may be associated with biofilm formation (Dong *et al.*, 2008). To investigate the effect of the *spiA* gene on biofilm formation by *S. enteritidis*, a *spiA* mutant was constructed, and the biological characteristics of the mutant and wild-type were compared. In a quantitative microtitre assay, the OD_600_ of Δ*spiA* was significantly lower than that of the wild-type strain, C50041, which is consistent with previous observations (Dong *et al.*, 2008). Both Δ*spiA* and C50041 can form biofilms on flask walls, but the biofilm formed by Δ*spiA* was washed off from the flask wall easily when it was stained by crystal violet. Furthermore, a *spiA* complementation strain regained the same biofilm characteristics as the wild-type strain. Scanning electron microscopy showed that the Δ*spiA* strain grown on polystyrene coverslips produced a loose and thin biofilm structure with fewer bacteria, while C50041 produced a very tenacious and thick structure with extracellular substances and fibrils. These results indicated that deletion of the *spiA* gene resulted in a deficiency in biofilm formation.

The morphotypes of *Salmonella* grown on Congo red and Coomassie brilliant blue G agar were grouped into five categories: (1) red, dry and rough, indicating curli and cellulose production (RDAR); (2) brown, dry and rough, indicating a lack of cellulose synthesis (BDAR); (3) pink, dry and rough, indicating a defect in curli expression (PDAR); (4) smooth, brown and mucoid, indicating a lack of cellulose synthesis but overproduced capsular polysaccharide (SBAM); and (5) smooth and white, indicating a lack of both curli and cellulose production (SAW) (Malcova *et al.*, 2008; Römling, 2005; Solomon *et al.*, 2005). The Δ*spiA* strain appeared to be a morphotype of RDAR and had a similarly bright fluorescence to C50041, indicating the presence of curli and cellulose production. However, Δ*spiA* in the biofilm phase showed a significant decrease in curli production, and a *spiA* complementation strain in the biofilm phase showed a similar amount of curli production to the wild-type strain. Because curli seem to be more important than cellulose for the formation of cell aggregates (Jonas *et al.*, 2007), the reduced expression of curli in Δ*spiA* may result in deficient biofilm formation.

It has been reported that a *spiA* mutant of *S. typhimurium* displayed wild-type levels of epithelial cell invasion but was defective for intramacrophage survival (Ochman *et al.*, 1996). In our study, regardless of the phase of growth the bacteria were in, Δ*spiA* showed similar adherence to and invasion of epithelial cells as compared with the wild-type strain. In contrast, Δ*spiA* showed intracellular proliferation in macrophages in both the exponential growth and the biofilm phases; this may have been due to the use of a different macrophage cell line. Interestingly, Δ*spiA* and the wild-type strain in the biofilm phase showed much greater adherence to and invasion of epithelial cells or intracellular proliferation in macrophages than those in the exponential growth phase. However, Δ*spiA* in the biofilm phase showed significantly lower intracellular proliferation than the wild-type strain. Consistent with previous reports (Miki *et al.*, 2004; Ochman *et al.*, 1996), Δ*spiA* did not kill BALB/c mice when inoculated intraperitoneally at >10^5^ times the median lethal dose of the wild-type strain. In addition, the LD_50_ in mice with Δ*spiA* in the biofilm phase was higher than that in mice with Δ*spiA* in the exponential growth phase; and the LD_50_ in mice with the wild-type in the biofilm phase was lower than that in mice with the wild-type in the exponential growth phase, indicating that deletion of the *spiA* gene resulted in a deficiency in biofilm formation and virulence. With regard to the bacterial distribution in mouse organs, Δ*spiA* showed a similar invasion rate in blood and other organs as the wild-type strain at 6 h post-challenge, but a significantly lower bacterial number in the blood, liver, spleen and lungs at 48 h post-challenge. Consistent with the results of bacterial adherence to and invasion of epithelial cells and bacterial intramacrophage proliferation, bacteria in the biofilms were more invasive in the mouse model than were bacteria in the exponential growth phase. Although the numbers of bacteria of the wild-type strain in both the exponential growth and the biofilm phases showed increases in blood, liver, spleen and lungs from 6 to 48 h post-infection, numbers of Δ*spiA* bacteria in the biofilm phase showed an accelerated clearance when compared with that of Δs*piA* in the exponential growth phase. An additional experiment confirmed that both Δ*spiA* and the wild-type strain were not sensitive to the normal BALB/c mouse serum (data not shown). The result indicated that deletion of *spiA* in *S. enteritidis* had no effect on its adherence and invasion ability, but may result in easy clearance by host cells. Given that the SpiA protein plays a major role in significantly reduced virulence (Ochman *et al.*, 1996), the mechanisms of virulence in bacterial biofilms need further study.

In conclusion, we have constructed a *spiA* mutant of *S. enteritidis* and showed that it is deficient in biofilm formation. Although the lack of *spiA* had no effect on adherence and invasion in epithelial cells, the Δ*spiA* mutant in both exponential growth and biofilm phases showed reduced adherence and invasion in a mouse model.
